# Domingos Edgardo Junqueira de Moraes 19/09/1921 - 30/07/2019

**DOI:** 10.21470/1678-9741-2019-0607

**Published:** 2019

**Authors:** Milton A. Meier

**Affiliations:** 1 Member of the Academia Nacional de Medicina, Rio de Janeiro, RJ, Brazil.

**Figure:**
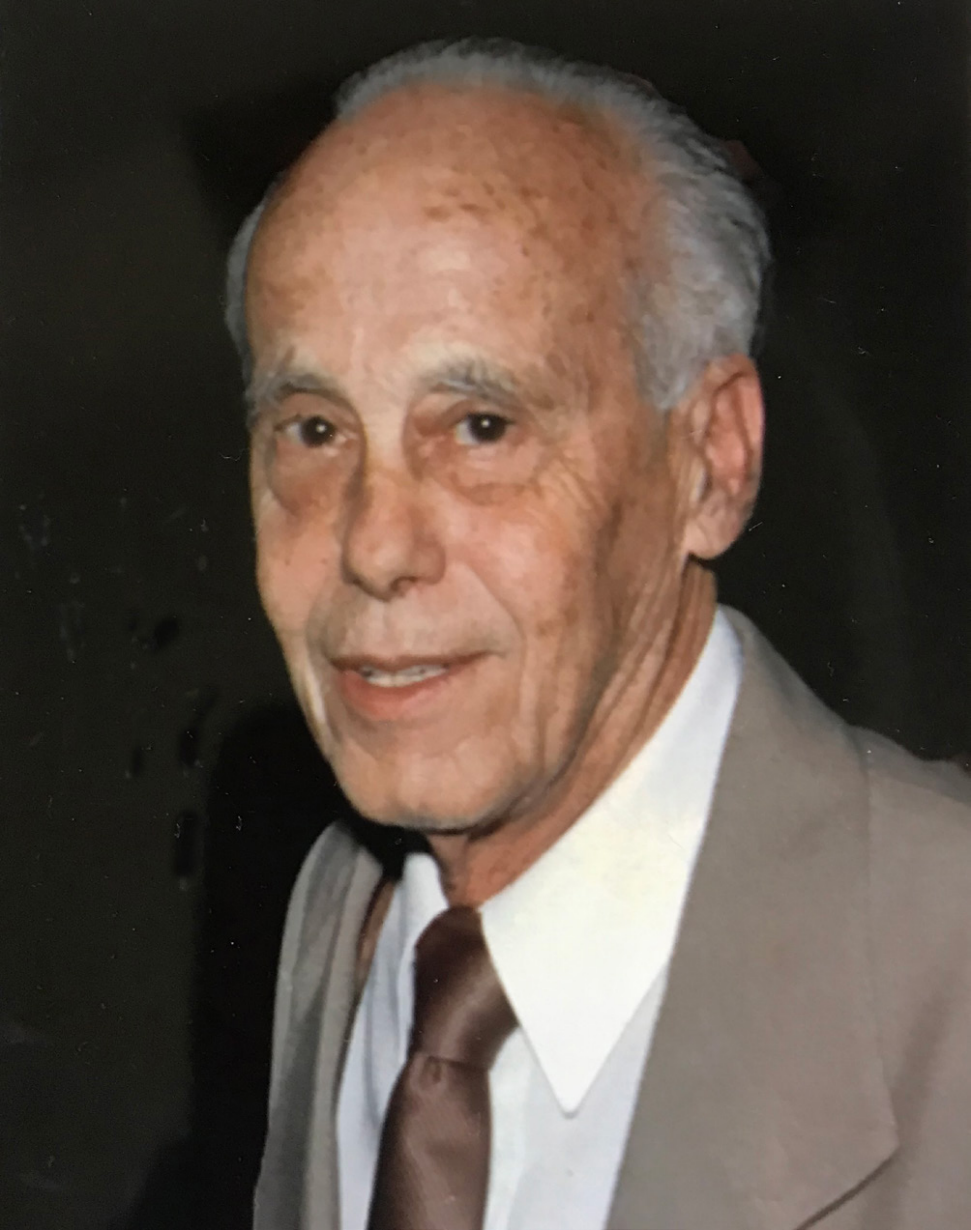
Domingos Edgardo Junqueira de Moraes

In early 1938, a thin and frail 17-year-old boy arrived in Rio de Janeiro, coming from the small town of Carmo de Minas, in the southern part of Minas Gerais.

Fatherless since two years old, he was full of plans and dreams, but was in doubt if he should follow a path in medicine or engineering. At the last minute, he made his decision, and after being approved in the college entrance exams, he enrolled in the Brazil's National Faculty of Medicine, where he graduated as a doctor in 1948.

Less than 20 years later, Domingos Edgardo Junqueira de Moraes became the first Brazilian surgeon to perform an open-heart operation in Rio de Janeiro and the second in all Latin America.

Over the course of those two decades, Domingos became Fernando Paulino's first resident at the Casa de Saúde São Miguel, and he would go on to become a surgeon in what was then considered one of the best hospitals in the country.

He was admitted to the IAPC and began performing lung surgery with Professor Odorico Rocha.

In 1954, he attended the 2^nd^ World Congress of Cardiology in Washington DC., and was amazed by the round tables where Gross, Crafford, Blalock, Gibbon, and Lillehei presented the first sketches of a newborn specialty - cardiac surgery.

Leaving his wife and two young children behind, and on unpaid leave from two jobs, he went to the USA, where he spent a year and half in Philadelphia, with Robert Glover, and six months in Minnesota, commuting between Minneapolis, under the tutelage of Lillehei, and the Mayo Clinic, with Kirklin.

Domingos realized that the system used by Lillehei was more adequate for Brazil. He acquired the plans for DeWall's bubble oxygenator and bought a Sigmamotor pump financed by his boss, Fernando Paulino.

Back in Brazil, he assembled a bubble oxygenator and began experimenting with it in dogs. He operated on 19 animals with a survival rate of over 80%, then he decided that the next operation would be on a human patient. On September 23, 1957, he successfully operated a 31-year-old patient suffering from a VSD. In that year, he operated four more patients, one of them underwent a Fallot Tetralogy correction, and all procedures were successful.

In the early stages of these experimental surgical methods, Domingos began to add saline to the prime volume of the oxygenator and the pump system. He noted that the survival rates in animals remained the same. Thus, he began to dilute the filling volume of patients with saline. It was a brilliant idea, which became known as ‘hemodilution’. He published his invention in the Revista Brasileira de Cirurgia (Brazilian Journal of Surgery) in 1960. Soon after that, others had the same idea; Zuhdi, in Oklahoma, and Cooley, in Houston, also published studies on hemodilution in 1962 in widely circulated medical publications.

In 1964, Domingos and his team moved to the Silvestre Hospital. There they had room to expand their activities. Taking advantage of the hospital's workshop, Domingos, Waldir Jazbik, and a mechanic called Gilberto Santos built a roller pump and created a stainless steel bubble oxygenator to replace the outdated DeWall's helix oxygenator. In his first month at the new hospital, Domingos implanted a Starr-Edwards prosthesis to replace a mitral valve ravaged by rheumatic disease. Soon after that, he did the same thing on an aortic valve. Both procedures were the first of their kind in Rio de Janeiro and one of the firsts in Brazil. In 1965, he was also the first to install an implantable pacemaker in Rio de Janeiro.

Domingos was a man who never took his victories and achievements for granted. He was an innovator and he was always open to suggestions. In addition to being the first Brazilian surgeon to adopt the bubble oxygenator, he soon began to associate hypothermia with cardiopulmonary bypass. He was among the first surgeons to adopt arterial return directly into the aorta, rather than return it through the femoral artery. Between 1961 and 1968, 13 patients were operated with cardiopulmonary bypass during their pregnancy, and no patient or fetus was lost. This was the subject of several congress presentations and a doctoral dissertation at the University of Brazil.

The Silvestre Hospital became a center of attraction thanks to the cardiac surgery achievements of Domingos Junqueira de Moraes. It was a hospital visited by surgeons from all over Brazil, who came in search of knowledge and training. Domingos was a mentor for a large number of surgeons who currently occupy leading positions in various states of our country.

In the 1970's, having brought a disposable Bentley oxygenator from the USA, he began to manufacture and market a similar device in Brazil. Domingos, in Rio de Janeiro, along with Adib Jatene, in São Paulo, and Domingo Braile, in São José do Rio Preto, were responsible for kick-starting the Brazil's industry of surgical equipment and products, which made it possible for Brazilian cardiology to compete on an equal footing with other countries.

Professor Domingos was always very creative, and in 1997 he published in the Brazilian Journal of Cardiovascular Surgery (BJCVS) an article^[1]^ with a new concept in the extracorporeal circulation. The article’s title was ‘Use of pure oxygen and venous-arterial shunt in membrane oxygenators’. The hypothesis was to avoid all the venous blood to pass through the oxygenator, shunting the venous line to the arterial line, avoiding the contact of the blood with the surface of the oxygenator, and using only oxygen instead of a mixture of oxygen and air. Surely the amount of blood to be shunted was determined by the oxygen saturation in the arterial line. This was the normal behavior of Dr. Moraes, permanently trying to simplify the cardiac surgery.

Over the course of his career, he participated frequently in surgery, cardiac surgery, and cardiology congresses, both in Brazil and abroad, presenting works, giving conferences, and as a member of round tables. He was a Professor at the then Faculdade Nacional de Medicina da Universidade do Brasil, today UFRJ, a Full Professor of Cardiovascular Surgery at the Universidade Federal Fluminense, a Full Member, and then Emeritus, of the Colégio Brasileiro de Cirurgiões, a Full Member of the Sociedade Brasileira de Cirurgia Cardiovascular, a Full Member of the Sociedade Brasileira de Cardiologia, and a recipient of the Order of Medical Merit.

Domingos was a surgeon who regarded his profession as a mission, and he knew well his place in the history of cardiac surgery in Brazil. Outside of the hospital, he was a kind, talkative, and intelligent man, who was always up to date with things. There was a certain folklore about his forgetfulness and confusion, but he embraced these anecdotes and even fed some of the stories. He liked to sit in a bar, have a good beer, and a good chat.

For almost five decades, Domingos was married to Alda Coli Junqueira, a descendant of Italians and daughter of a country doctor. They had four children: Mario Coli Junqueira, a surgeon like his father, Zuleica Junqueira de Moraes, a cardiologist, Mônica, and Domingos.

Domingos retired in 1991 and when he stopped operating, gradually he moved away from the profession he so loved and to which he dedicated most of his life. Time unmercifully reduced his hearing, and out of stubbornness, he refused to wear hearing aids. Time also blurred his vision. With that, he isolated himself from the conversations he enjoyed so much and remained silent.

In 2007, he lost his wife, which was a terrible blow for him. Soon after that, he lost his closest brother, Henrique. For Domingos Junqueira de Moraes, the light went out on the afternoon of the 30^th^ day of last July.

From now on, he occupies a place in the pantheon reserved for the greats. Today we mourn Domingos' loss, but we celebrate the almost 100 years he lived among us.
